# Saliency-Guided Nonsubsampled Shearlet Transform for Multisource Remote Sensing Image Fusion

**DOI:** 10.3390/s21051756

**Published:** 2021-03-04

**Authors:** Liangliang Li, Hongbing Ma

**Affiliations:** Department of Electronic Engineering, Tsinghua University, Beijing 100084, China; leeliangliang@tsinghua.edu.cn

**Keywords:** multisource remote sensing image, image fusion, contrast saliency map, SML, NSST

## Abstract

The rapid development of remote sensing and space technology provides multisource remote sensing image data for earth observation in the same area. Information provided by these images, however, is often complementary and cooperative, and multisource image fusion is still challenging. This paper proposes a novel multisource remote sensing image fusion algorithm. It integrates the contrast saliency map (CSM) and the sum-modified-Laplacian (SML) in the nonsubsampled shearlet transform (NSST) domain. The NSST is utilized to decompose the source images into low-frequency sub-bands and high-frequency sub-bands. Low-frequency sub-bands reflect the contrast and brightness of the source images, while high-frequency sub-bands reflect the texture and details of the source images. Using this information, the contrast saliency map and SML fusion rules are introduced into the corresponding sub-bands. Finally, the inverse NSST reconstructs the fusion image. Experimental results demonstrate that the proposed multisource remote image fusion technique performs well in terms of contrast enhancement and detail preservation.

## 1. Introduction

Remote sensing images play an important role in urban planning, environmental monitoring, and military defense [1]. As a basic step of target classification, detection, and recognition in remote sensing images, remote sensing image fusion has attracted more and more research interest across the world. Due to the incident wavelengths of the remote sensing images in the same region being different, multiband remote sensing images have significant differences. The high-band remote sensing image can provide an overall view of the scene, which is similar to optical imaging, while the low-band remote sensing image is relatively bleak and has deeper penetration. Remote sensing image fusion can integrate multiband remote sensing images into a comprehensive image, which is conducive to the recognition and observation of ground objects [1,2,3].

Multisource remote sensing image fusion is an information processing technology for the fusion of multisensor, multiplatform remote sensing and multispectral band remote sensing data. The fusion image contains different spatial, temporal, and spectral information of multisensor, which allows for preparation for further analysis and processing. Many image fusion methods have been proposed in recent decades; however, image fusion algorithms based on transform domain and edge-preserving filters are widely used [4]. In terms of transform domain-based image fusion frameworks, the wavelet transform, discrete wavelet transform (DWT) [5], dual-tree complex wavelet transform (DTCWT) [5], dual-tree complex wavelet package transform (DTCWPT) [6], framelet transform [7], curvelet transform [5], contourlet transform [8], nonsubsampled contourlet transform (NSCT) [9], shearlet transform [10], and nonsubsampled shearlet transform (NSST) [11], etc., are adapted to the field of image fusion. Iqbal et al. [12] introduced a multifocus image fusion approach using a DWT and a guided image filter to improve the definition of the fused images. Aishwarya et al. [13] used a DTCWT and an adaptive combined clustered dictionary for visible and infrared image fusion to enhance the target information. Wang et al. [14] proposed a multispectral (MS) and panchromatic (PAN) image fusion technique based on the hidden Markov tree model in a complex tight framelet transform domain to improve the spatial resolution of the MS image while keeping the spectral information. Due to the fact that the wavelet transform cannot capture the abundant directional information of remote sensing images and can introduce spatial distortion, a contourlet transform and an NSCT are introduced to resolve this shortcoming. Yang et al. [15] proposed a remote sensing image fusion algorithm via a contourlet hidden Markov tree and a clarity–saliency-driven pulse couple neural network (PCNN) model to enhance the edges and contours of fused remote sensing images. Li et al. [16] introduced an image fusion method using dynamic threshold neural P systems and NSCT for multimodality medical imaging to improve the visual quality and fusion performance. Because the contourlet transform- and NSCT-based image fusion approaches are computationally complex, the shearlet transform and the NSST are proposed to increase computational efficiency. Because the shearlet transform lacks translation invariance, the NSST has become more widely used as the improved version of the shearlet transform in the field of image processing. Yin et al. [17] proposed an image fusion technique via NSST and parameter-adaptive pulse coupled neural network (PAPCNN) to improve the contrast and brightness of the fused medical images. Wang et al. [18] introduced the nonsubsampled shearlet transform hidden Markov forest (NSST-HMF) model for pansharpening to improve the spatial resolution of hyperspectral images while preserving spectral features.

In terms of edge preserving filter-based image fusion approaches, the guided image filter, cross bilateral filter, and rolling guidance filter, etc., are widely used. Li et al. [19] first introduced the guided image filter for image fusion, for which the computational complexity is relatively low. Then, the combination of guided image filtering and other transform domain algorithms such as DTCWT, NSCT, and NSST is introduced into the field of image fusion, and good results are achieved. Shreyamsha et al. [20] introduced the cross bilateral filter for image fusion based on pixel significance to enhance the visual quality of the fused images. Jian et al. [21] proposed a multiscale image fusion method using a rolling guidance filter to preserve the details and suppress the artifacts of the fused images.

In this work, a novel remote sensing image fusion algorithm using a contrast saliency map (CSM) and SML in the NSST domain is proposed. The contrast saliency map-based fusion rule and SML-based fusion rule are used to merge the low- and high-frequency sub-bands, respectively. Experimental results demonstrate the effectiveness of the proposed remote sensing image fusion method over the traditional and state-of-the-art fusion algorithms in terms of qualitative and quantitative analysis.

The rest of this work is organized as follows: Section 2 shows the related works, Section 3 depicts the proposed remote sensing image fusion method, the experiments and results are summarized in Section 4, and the conclusions are provided in Section 5.

## 2. Related Works

### Nonsubsampled Shearlet Transform

**Figure 1:**
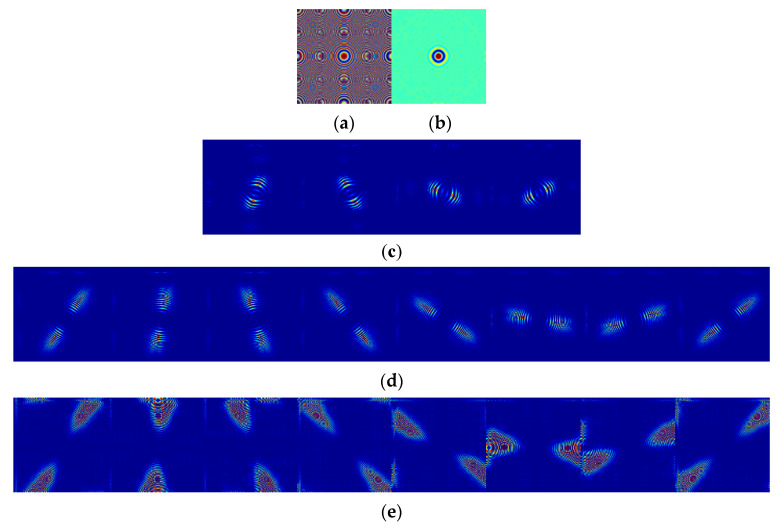
The nonsubsampled shearlet transform (NSST) decomposition of a zoneplate image. (**a**) original zoneplate image, (**b**) the low-frequency component, (**c**) the high-frequency sub-bands of NSST decomposition at level 1, (**d**) the high-frequency sub-bands of NSST decomposition at level 2, (**e**) the high-frequency sub-bands of NSST decomposition at level 3.

Nonsubsampled shearlet transform (NSST) is a kind of nonsubsampled multiscale transform, which was introduced based on the theory of shearlet transform [11,18]. The image is decomposed by NSST into multiple scales with multiple directions by multiscale and multidirectional decompositions. Firstly, the nonsubsampled pyramid (NSP) is adopted as the multiscale decomposition filter to decompose the image into one low-frequency sub-band and one high-frequency sub-band. Then, the high-frequency sub-band is decomposed by the shearing filter (SF) to achieve the multidirectional sub-bands. Due to the NSST decomposition process having no subsampling for the NSP and the SF, the NSST is shift-invariant. Figure 1 denotes the example of three levels of NSST decomposition of a zoneplate image, where all the images are displayed in the “jet” colormap and the direction numbers from coarser to finer are 4, 8, and 8. Figure 1a depicts the original zoneplate image, Figure 1b shows the low-frequency component, and Figure 1c–e show the high-frequency sub-band images with the direction numbers 4, 8, and 8, respectively.

## 3. Proposed Fusion Method

**Figure 2:**
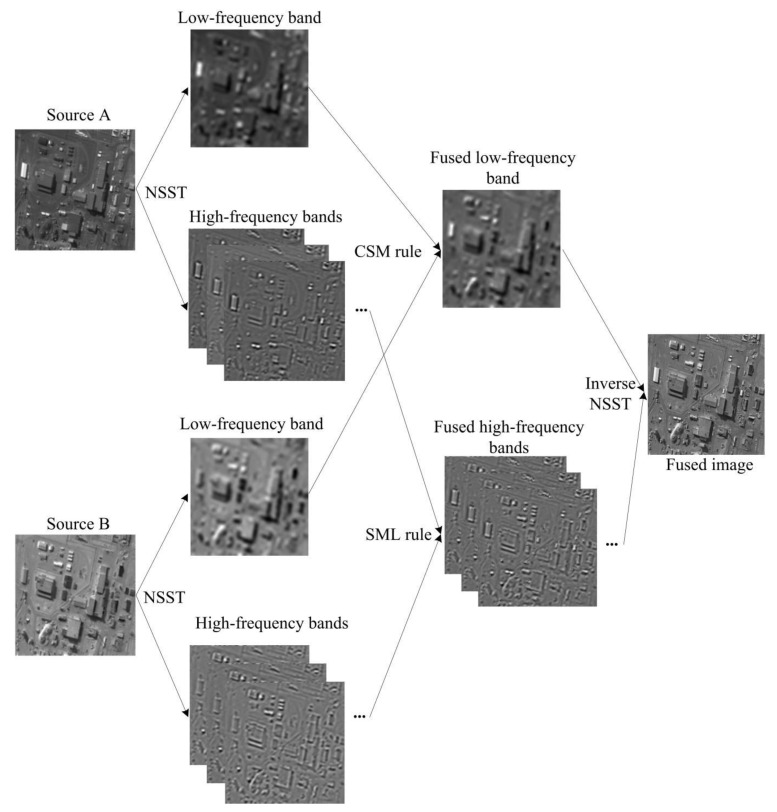
The flow chart of the proposed remote sensing image fusion method.

In this section, a novel remote sensing image fusion method based on the NSST is proposed, and the whole process can be divided into four parts: NSST decomposition, low-frequency sub-band fusion, high-frequency sub-band fusion, and inverse NSST image reconstruction. Suppose the input remote sensing images are *A* and *B*, then the two images are decomposed up to *N* levels utilizing the NSST to generate the decomposed sub-bands $\left\{L_{A},H_{A}^{l,d}\right\}$ and $\left\{L_{B},H_{B}^{l,d}\right\}$, respectively. The $H_{X}^{l,d}|X\in \left\{A,B\right\}$ represents the high-frequency sub-bands of *X* achieved at the *l*th decomposition with the direction *d*, the $L_{X}|X\in \left\{A,B\right\}$ represents the low-frequency sub-band of *X*, where l∈[1,N], d∈[1,D(l)], *N* equals the number of NSST decomposition levels, and *D* denotes the vector which concludes the number of directions at each *l*. The fused image *F* is generated by inverse NSST performed on the fused sub-bands $\left\{L_{F},H_{F}^{l,d}\right\}$. The flow chart of the proposed remote sensing image fusion approach is shown in Figure 2. The fusion rules for low-frequency and high-frequency components are summarized as follows.

### 3.1. Fusion of Low-Frequency Components

The low-frequency sub-bands present the brightness and contrast information of the source remote sensing images [22]. In this section, in order to preserve the contrast, the contrast saliency maps (CSM) of the low-frequency components are constructed based on the brightness distribution. The contrast of the image denotes the difference between the lowest and highest brightness levels in the remote sensing images, and where the difference in brightness is more significant, a higher contrast is implied. Therefore, we can infer that the brighter or darker the pixel value is relative to the average value of the image, the greater its contribution to the image contrast and the stronger the contrast significance. The *L_2_* norm is used to judge the deviation between pixel value and average value, and the significance of each pixel is expressed. When the *L*_2_ norm is performed on the low-frequency sub-bands *L_A_* and *L_B_*, the contrast saliency maps $S_{L_{A}}$ and $S_{L_{B}}$ of the low-frequency sub-bands are generated by the following:

(1) $$S_{L_{A}}=norm\left({\left\|L_{A}-mean\left(L_{A}\right)\right\|}_{2}\right)$$

(2) $$S_{L_{B}}=norm\left({\left\|L_{B}-mean\left(L_{B}\right)\right\|}_{2}\right)$$

where the mean(⋅) denotes the average value of the image. *L*_2_ norm is used to eliminate the effect of symbols, and the norm(⋅) function is defined as follows:

(3) $$norm\left(x\right)=\frac{x-min\left(x\right)}{max\left(x\right)-min\left(x\right)}$$

The weight matrices $W_{L_{A}}$ and $W_{L_{B}}$ of the low-frequency components are calculated by the following formulas performed on the saliency maps of the low-frequency sub-bands:

(4) $$W_{L_{A}}=0.5+0.5\left(S_{L_{A}}-S_{L_{B}}\right)$$

(5) $$W_{L_{B}}=0.5+0.5\left(S_{L_{B}}-S_{L_{A}}\right)$$

The fused low-frequency sub-bands are computed by the Hadamard product performed on the low-frequency components and the corresponding weight matrices, and the corresponding equation is defined as follows:

(6) $$L_{F}=W_{L_{A}}*L_{A}+W_{L_{B}}*L_{B}$$

where $L_{F}$ represents the fused low-frequency component, and * shows the Hadamard product.

### 3.2. Fusion of High-Frequency Components

The high-frequency components contain the texture information and details. In this section, the sum-modified-Laplacian (SML) is used to process the high-frequency sub-bands. The SML is defined for the local window with the size (2P+1)(2Q+1), and the corresponding formula is calculated by [23]:

(7) $$SML^{l,d}\left(i,j\right)=\sum_{p=-P}^{P}\sum_{q=-Q}^{Q}{\left[ML^{l,d}\left(i+p,j+q\right)\right]}^{2}$$

(8) $$\begin{array}{cc} ML^{l,d}\left(i,j\right)= & \left|2H^{l,d}\left(i,j\right)-H^{l,d}\left(i-step,j\right)-H^{l,d}\left(i+step,j\right)\right|\\ & +\left|2H^{l,d}\left(i,j\right)-H^{l,d}\left(i,j-step\right)-H^{l,d}\left(i,j+step\right)\right| \end{array}$$

where *step* denotes the changeable interval among the high-frequency coefficients. It is usually defined as 1.

The fused high-frequency sub-bands can be computed by:

(9) $$H_{F}^{l,d}\left(i,j\right)=\left\{ \begin{array}{l} H_{A}^{l,d}\left(i,j\right)\;\mathrm{if}\;SML_{A}^{l,d}\left(i,j\right)\geq SML_{B}^{l,d}\left(i,j\right)\\ H_{B}^{l,d}\left(i,j\right)\;\mathrm{if}\;SML_{A}^{l,d}\left(i,j\right)<SML_{B}^{l,d}\left(i,j\right) \end{array}\right.$$

where *H_F_* denotes the fused high-frequency components.

The whole procedure of the proposed remote sensing image fusion method can be summarized in Algorithm 1.
**Algorithm 1** Remote sensing image fusion via NSST**Input:** the source remote sensing images *A* and *B*
**Output:** fused image *F*
**Parameters:** the number of NSST decomposition levels—*N*; the number of directions at each decomposition level—D(l),l∈[1,N]
**Step 1:** NSST decomposition The input images *A* and *B* are decomposed into low- and high-frequency sub-bands $\left\{L_{A},H_{A}^{l,d}\right\}$ and $\left\{L_{B},H_{B}^{l,d}\right\}$, respectively. **Step 2:** low-frequency band fusion rule (1) The saliency maps $\left(S_{L_{A}},S_{L_{B}}\right)$ and the corresponding weight matrices $\left(W_{L_{A}},W_{L_{B}}\right)$ of the low-frequency bands are calculated by Equations (1)–(5). (2) The fused low-frequency band *L_F_* is obtained by Equation (6). **Step 3:** high-frequency band fusion rule (1) The *SML* of the high-frequency bands is constructed via Equations (7)–(8). (2) The fused high-frequency band *H_F_* is computed by Equation (9). **Step 4:** inverse NSST and image reconstruction The fused image *F* is reconstructed by inverse NSST performed on the fused low- and high-frequency bands $\left\{L_{F},H_{F}^{l,d}\right\}$.

## 4. Experimental Results and Discussion

**Figure 3:**
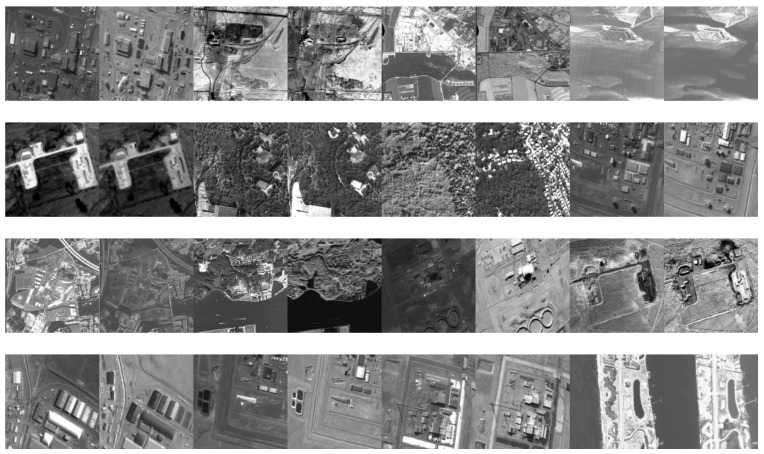
Multisource remote sensing image data sets.

In this section, in order to demonstrate the effectiveness of the proposed multisource remote sensing image fusion method via NSST, public data sets (https://sites.google.com/view/durgaprasadbavirisetti/datasets (accessed on 15 December 2020)) are used for simulation, and several state-of-the-art image fusion algorithms are adapted for comparison, namely image fusion based on a guided image filter (GFF) [19], image matting for the fusion of multifocus image (IFM) [24], image fusion using a dual-tree complex wavelet transform (DTCWT) [5], curvelet transform-based image fusion (CVT) [5], image fusion utilizing phase congruency (PC) [25], structure-aware image fusion (SAIF) [26], fusing infrared and visible images of different resolutions via total variation model (DRTV) [27], multimodal image seamless fusion (MISF) [28], and parameter-adaptive pulse-coupled neural network-based image fusion via a nonsubsampled shearlet transform (NSST) [17]. In order to reflect the fairness of the algorithm, the parameters of the comparison algorithms are consistent with the original published papers. In the proposed fusion technique, the number of NSST decomposition levels is four, and the direction numbers from coarser to finer are 8, 8, 16, and 16. The selected remote sensing image data sets are shown in Figure 3.

**Figure 4:**
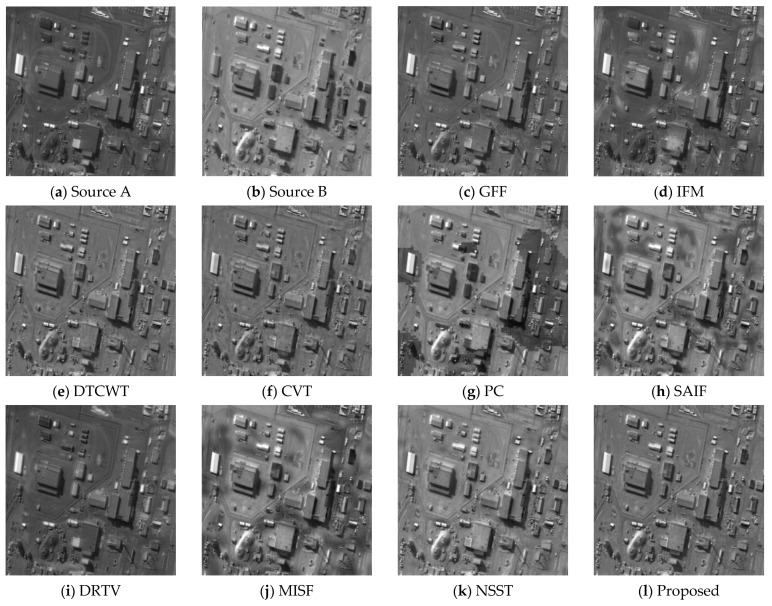
Fusion results of the first group of images. (**a**) Source A, (**b**) Source B, (**c**) guided image filter (GFF), (**d**) image matting for fusion (IFM), (**e**) dual-tree complex wavelet transform (DTCWT), (**f**) curvelet transform-based image fusion (CVT), (**g**) phase congruency (PC), (**h**) structure-aware image fusion (SAIF), (**i**) different resolutions via total variation (DRTV), (**j**) multimodal image seamless fusion (MISF), (**k**) nonsubsampled shearlet transform (NSST), (**l**) proposed method.

**Figure 5:**
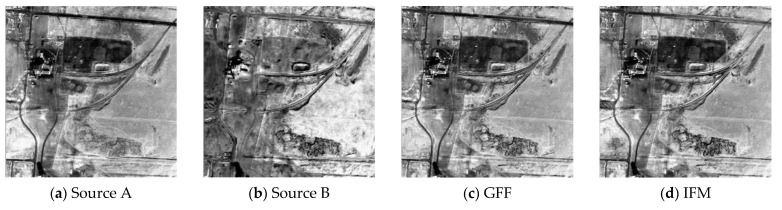

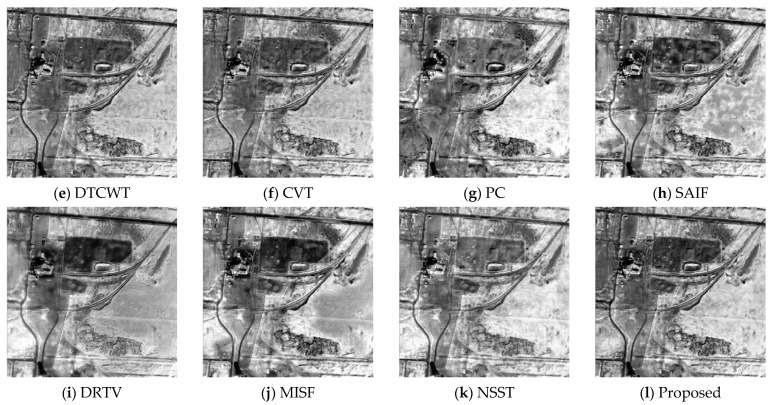
Fusion results of the second group of images. (**a**) Source A, (**b**) Source B, (**c**) GFF, (**d**) IFM, (**e**) DTCWT, (**f**) CVT, (**g**) PC, (**h**) SAIF, (**i**) DRTV, (**j**) MISF, (**k**) NSST, (**l**) proposed method.

**Figure 6:**
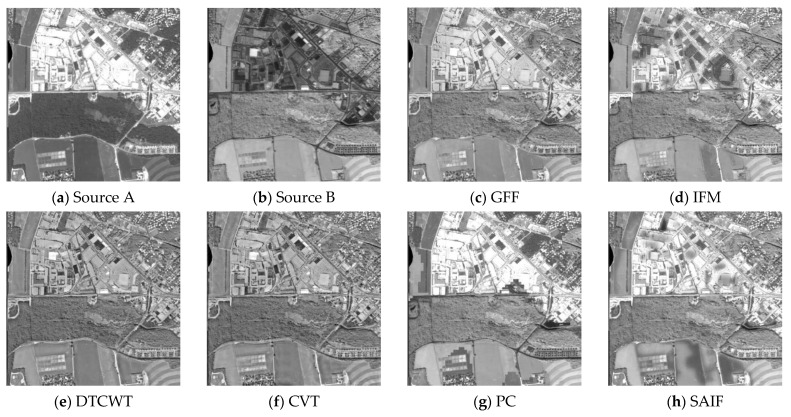

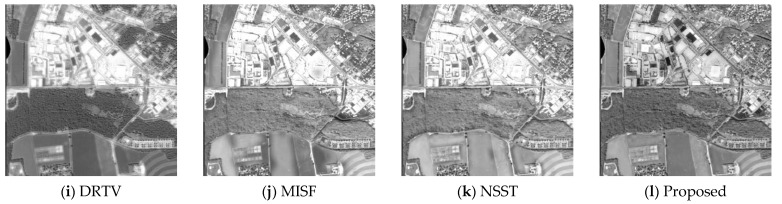
Fusion results of the third group of images. (**a**) Source A, (**b**) Source B, (**c**) GFF, (**d**) IFM, (**e**) DTCWT, (**f**) CVT, (**g**) PC, (**h**) SAIF, (**i**) DRTV, (**j**) MISF, (**k**) NSST, (**l**) proposed method.

**Figure 7:**
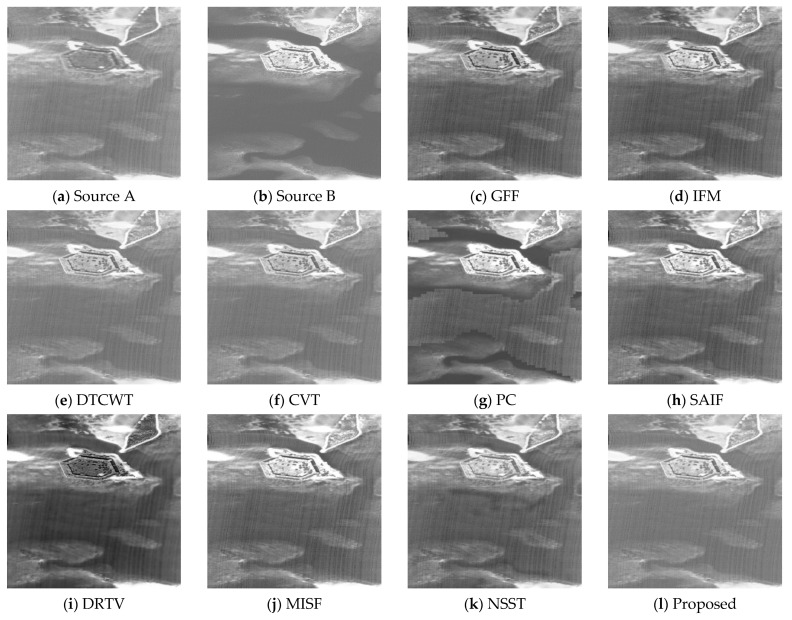
Fusion results of the fourth group of images. (**a**) Source A, (**b**) Source B, (**c**) GFF, (**d**) IFM, (**e**) DTCWT, (**f**) CVT, (**g**) PC, (**h**) SAIF, (**i**) DRTV, (**j**) MISF, (**k**) NSST, (**l**) proposed method.

In order to objectively assess the fusion performances of all the different fusion techniques, a lot of image fusion evaluation indexes have been introduced in these years. It is known to us that just one evaluation index could not well demonstrate the quality of fused images in quantitative assessment. Thus, for the sake of making a comprehensive evaluation for the fusion images, six popular fusion evaluation metrics are introduced in this section, namely visual information fidelity for fusion (VIFF) [29,30,31,32,33], Q_S_ [34], average gradient (AG) [20,35,36], correlation coefficient (CC) [20,37,38], spatial frequency (SF) [20,39,40,41], and Q_W_ [34,42]. In terms of all the six metrics, the higher the value data of the evaluation index, the better the fusion performance will be. The experimental results are depicted in Figure 4, Figure 5, Figure 6 and Figure 7 and Table 1, Table 2, Table 3, Table 4 and Table 5.

### 4.1. Qualitative Analysis

In this section, the fusion results obtained by the proposed method and the compared results calculated by nine other fusion algorithms are given in Figure 4, Figure 5, Figure 6 and Figure 7. The Figure 4, Figure 5, Figure 6 and Figure 7a,b show the source images A and B, respectively. As seen from Figure 4, the GFF, DTCWT, CVT and DRTV algorithms decrease the contrast of the fusion images, making some details invisible (see Figure 4c,e,f,i). The IFM, SAIF, and MISF methods appear to generate a block effect and artifacts, affecting the observation of the fused images (see Figure 4d,h,j). The PC algorithm makes the image distorted (see Figure 4g). The NSST technique provides overly high brightness (see Figure 4k). The proposed fusion technique can provide a high-definition image and preserve spatial detail information in the fused image (see Figure 4l).

From Figure 5, we can see that the GFF, IFM, and DRTV methods make the fused image darker in some regions (see Figure 5c,d,i). The DTCWT and CVT methods make the fused images better compared to the previous methods (see Figure 5e,f). The PC approach provides a poor fusion performance (see Figure 5g). The SAIF and MISF algorithms introduce artifacts (see Figure 5h,j). The NSST method makes the fused image brighter, and it is not conducive to the acquisition of target information from the fused image (see Figure 5k). The proposed fusion method provides a better fusion effect (see Figure 5l).

From Figure 6, it can be seen that the GFF, IFM, DTCWT, and CVT algorithms decrease the contrast and make the images darker (see Figure 6c–f). The PC technique appears to generate a block effect (see Figure 6g). The SAIF, MISF, and NSST methods produce artifacts, and the brightness is over-enhanced in some regions (see Figure 6h,j,k). The DRTV method produces over-enhanced brightness in some regions and an overly smooth fusion image (see Figure 6i). The proposed algorithm can enhance the contrast and definition, which is helpful in obtaining the target information from the fused image (see Figure 6l).

From Figure 7, we can see that the GFF, IFM, SAIF, and MISF algorithms make the fusion image darker (see Figure 7c,d,h,j). The DTCWT and CVT methods produce a good fusion visual effect (see Figure 7e,f). The PC, DRTV, and NSST techniques produce distortion and artifacts (see Figure 7g,i,k). The proposed fusion technique can produce relatively higher contrast and preserve the texture information (see Figure 7l).

In summary, the analysis of the subjective assessment of the fusion results demonstrates the super-performance of the proposed remote sensing image fusion technique when compared with the state-of-the-art fusion algorithms.

### 4.2. Quantitative Analysis

In this section, the six indexes (VIFF, Q_S_, AG, CC, SF, Q_W_) are used to evaluate the fusion results quantitatively. The data for the evaluation metrics of the different fusion algorithms for Figure 4, Figure 5, Figure 6 and Figure 7 are shown in Table 1, Table 2, Table 3 and Table 4. From Table 1, we can see that the value of VIFF as computed by the proposed method is slightly worse than the NSST algorithm, while the data for the other five metrics as calculated by the proposed fusion technique are the best. From Table 2, we can see that the metric values given by the proposed method are the largest except for the metric of CC. From Table 3, the values of CC and Q_W_ as computed by the proposed technique are a little smaller than the corresponding values obtained by the CVT and NSST methods, respectively. From Table 4, we can see that all six values of the metrics achieved by the proposed method are the best compared to the other fusion methods.

**Figure 8:**
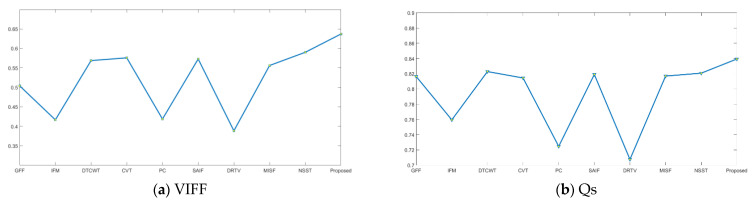

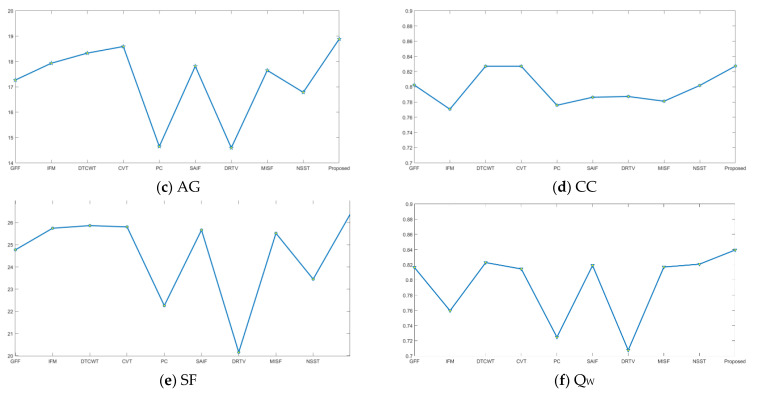
The line chart of objective metric data in Table 5. (**a**) VIFF; (**b**) Q_S_; (**c**) AG; (**d**) CC; (**e**) SF; (**f**) Q_W_.

In order to demonstrate the effectiveness of the proposed method, the sixteen image groups given in Figure 3 are simulated, and the average values of their objective evaluation are given in Table 5. The line charts of the objective metrics data in Table 5 are given in Figure 8, and the proposed method has the best values in the data for all metrics. Therefore, it is demonstrated that better fusion performance can be generated by the proposed remote sensing image fusion work.

## 5. Conclusions

In this work, a novel saliency-guided nonsubsampled shearlet transform for multisource remote sensing image fusion is introduced. First, the input images are transformed from the spatial domain to the shearlet domain according to a nonsubsampled shearlet transform. Second, the contrast saliency maps and corresponding weighted matrices are introduced for fusing the low-frequency coefficients, and the SML-based fusion rule is performed on the high-frequency coefficients, which can improve the contrast and definition of the fused images. To prove the universality of the proposed fusion algorithm, sixteen sets of remote sensing images are simulated, and six image fusion evaluation indexes are utilized for the quantitative analysis. From the experimental results, we can conclude that the proposed fusion approach has superior performance compared to the state-of-the-art fusion methods. In future work, we will extend the algorithm to panchromatic and multispectral [43,44,45,46,47,48], hyperspectral and multispectral image fusion [49,50].

## Figures and Tables

**Table 1:** The objective evaluation of the methods in Figure 4.

	VIFF	Q_S_	AG	CC	SF	Q_W_
GFF	0.4057	0.8064	8.7903	0.7493	14.4590	0.8079
IFM	0.2871	0.7174	9.5061	0.6834	15.6730	0.7091
DTCWT	0.5380	0.8140	10.0384	0.7816	15.7787	0.8214
CVT	0.5534	0.7984	10.2397	0.7771	15.5899	0.8165
PC	0.4246	0.7477	9.1494	0.6668	14.6779	0.6555
SAIF	0.5662	0.8038	9.3884	0.6798	15.2025	0.8261
DRTV	0.2895	0.7316	7.8006	0.7176	11.6689	0.6561
MISF	0.5226	0.8051	9.1365	0.6575	14.9136	0.8142
NSST	**0.6158**	0.8218	10.0766	0.7272	15.4583	0.8304
Proposed	0.6130	**0.8438**	**10.4592**	**0.7893**	**16.2149**	**0.8434**

**Table 2:** The objective evaluation of the methods in Figure 5.

	VIFF	Q_S_	AG	CC	SF	Q_W_
GFF	0.3982	0.7197	26.7401	**0.8926**	35.1380	0.7640
IFM	0.3679	0.6925	27.4735	0.8840	36.6562	0.7345
DTCWT	0.5255	0.7384	28.8500	0.8899	37.5651	0.7866
CVT	0.5396	0.7310	29.2726	0.8896	37.6290	0.7828
PC	0.3712	0.6379	24.6670	0.8748	34.9834	0.6894
SAIF	0.4689	0.7239	27.9649	0.8875	37.6971	0.7872
DRTV	0.3633	0.6082	22.4563	0.8694	31.2856	0.6744
MISF	0.4630	0.7252	27.2744	0.8859	36.6062	0.7721
NSST	0.5119	0.7521	28.8961	0.8820	37.0427	0.7872
Proposed	**0.5940**	**0.7625**	**30.1132**	0.8921	**38.9878**	**0.8034**

**Table 3:** The objective evaluation of the methods in Figure 6.

	VIFF	Q_S_	AG	CC	SF	Q_W_
GFF	0.4048	0.7965	22.7779	0.6300	33.9869	0.7602
IFM	0.2564	0.6778	23.4184	0.6315	34.6252	0.5919
DTCWT	0.4120	0.7772	24.5238	0.6583	35.9560	0.7537
CVT	0.4258	0.7614	24.8528	**0.6610**	35.6106	0.7490
PC	0.3381	0.7186	22.9823	0.6226	35.0967	0.6680
SAIF	0.3493	0.7689	24.1520	0.6217	36.0128	0.7543
DRTV	0.2970	0.6430	18.5259	0.5972	25.2082	0.5422
MISF	0.3838	0.7722	23.6538	0.6112	36.1746	0.7535
NSST	0.4299	0.7911	24.2249	0.6324	35.5451	**0.7750**
Proposed	**0.5430**	**0.7965**	**25.3122**	0.6512	**36.5362**	0.7706

**Table 4:** The objective evaluation of the methods in Figure 7.

	VIFF	Q_S_	AG	CC	SF	Q_W_
GFF	0.7339	0.9520	13.4416	0.9325	17.0349	0.9294
IFM	0.6886	0.9465	13.5312	0.9302	17.1410	0.9100
DTCWT	0.7997	0.9497	13.7663	0.9413	17.6068	0.9306
CVT	0.8047	0.9485	13.8226	0.9409	17.5972	0.9304
PC	0.6968	0.8124	9.4584	0.8726	14.6077	0.8451
SAIF	0.7475	0.9510	13.2681	0.9320	17.0035	0.9297
DRTV	0.5262	0.6900	5.4341	0.9179	10.8994	0.7934
MISF	0.7429	0.9498	13.3593	0.9301	17.1603	0.9235
NSST	0.7133	0.9406	13.0894	0.9250	15.9954	0.9068
Proposed	**0.8260**	**0.9529**	**13.9189**	**0.9414**	**17.7991**	**0.9366**

**Table 5:** The average objective evaluation of the methods on the sixteen group images.

	VIFF	Q_S_	AG	CC	SF	Q_W_
GFF	0.5040	0.8165	17.2688	0.8025	24.7722	0.8166
IFM	0.4167	0.7596	17.9319	0.7706	25.7461	0.7344
DTCWT	0.5689	0.8229	18.3304	0.8271	25.8626	0.8266
CVT	0.5759	0.8145	18.5907	0.8271	25.8054	0.8230
PC	0.4188	0.7248	14.6469	0.7758	22.2573	0.6786
SAIF	0.5730	0.8191	17.8152	0.7863	25.6650	0.8366
DRTV	0.3885	0.7077	14.5927	0.7873	20.1573	0.6742
MISF	0.5563	0.8170	17.6502	0.7811	25.5196	0.8265
NSST	0.5902	0.8208	16.7840	0.8018	23.4510	0.8168
Proposed	**0.6372**	**0.8394**	**18.8870**	**0.8273**	**26.3930**	**0.8401**

## Data Availability

Not applicable.
